# Computed cardiopulmonography and the idealized lung clearance index, iLCI_2.5_, in early-stage cystic fibrosis

**DOI:** 10.1152/japplphysiol.00744.2022

**Published:** 2023-06-01

**Authors:** Dominic Sandhu, Jennifer L. Redmond, Nicholas M. J. Smith, Christopher Short, Clare J. Saunders, John H. Couper, Christopher J. Fullerton, Graham Richmond, Nick P. Talbot, Jane C. Davies, Grant A. D. Ritchie, Peter A. Robbins

**Affiliations:** ^1^Department of Chemistry, University of Oxford, Oxford, United Kingdom; ^2^Royal Brompton and Harefield Hospitals, Guys and St Thomas’ Trust, London, United Kingdom; ^3^National Heart and Lung Institute, Imperial College London, London, United Kingdom; ^4^European Cystic Fibrosis Society, Lung Clearance Index Core Facility, London, United Kingdom; ^5^Department of Physiology, Anatomy and Genetics, University of Oxford, Oxford, United Kingdom

**Keywords:** laser absorption spectroscopy, log-normal lung, lung function testing, multibreath washout, nitrogen washout

## Abstract

This study explored the use of computed cardiopulmonography (CCP) to assess lung function in early-stage cystic fibrosis (CF). CCP has two components. The first is a particularly accurate technique for measuring gas exchange. The second is a computational cardiopulmonary model where patient-specific parameters can be estimated from the measurements of gas exchange. Twenty-five participants (14 healthy controls, 11 early-stage CF) were studied with CCP. They were also studied with a standard clinical protocol to measure the lung clearance index (LCI_2.5_). Ventilation inhomogeneity, as quantified through CCP parameter σlnCl, was significantly greater (*P* < 0.005) in CF than in controls, and anatomical deadspace relative to predicted functional residual capacity (DS/FRCpred) was significantly more variable (*P* < 0.002). Participant-specific parameters were used with the CCP model to calculate idealized values for LCI_2.5_ (iLCI_2.5_) where extrapulmonary influences on the LCI_2.5_, such as breathing pattern, had all been standardized. Both LCI_2.5_ and iLCI_2.5_ distinguished clearly between CF and control participants. LCI_2.5_ values were mostly higher than iLCI_2.5_ values in a manner dependent on the participant’s respiratory rate (r = 0.46, *P* < 0.05). The within-participant reproducibility for iLCI_2.5_ appeared better than for LCI_2.5_, but this did not reach statistical significance (*F* ratio = 2.2, *P* = 0.056). Both a sensitivity analysis on iLCI_2.5_ and a regression analysis on LCI_2.5_ revealed that these depended primarily on an interactive term between CCP parameters of the form σlnCL*(DS/FRC). In conclusion, the LCI_2.5_ (or iLCI_2.5_) probably reflects an amalgam of different underlying lung changes in early-stage CF that would require a multiparameter approach, such as potentially CCP, to resolve.

**NEW & NOTEWORTHY** Computed cardiopulmonography is a new technique comprising a highly accurate sensor for measuring respiratory gas exchange coupled with a cardiopulmonary model that is used to identify a set of patient-specific characteristics of the lung. Here, we show that this technique can improve on a standard clinical approach for lung function testing in cystic fibrosis. Most particularly, an approach incorporating multiple model parameters can potentially separate different aspects of pathological change in this disease.

## Graphical abstract

**Figure:**
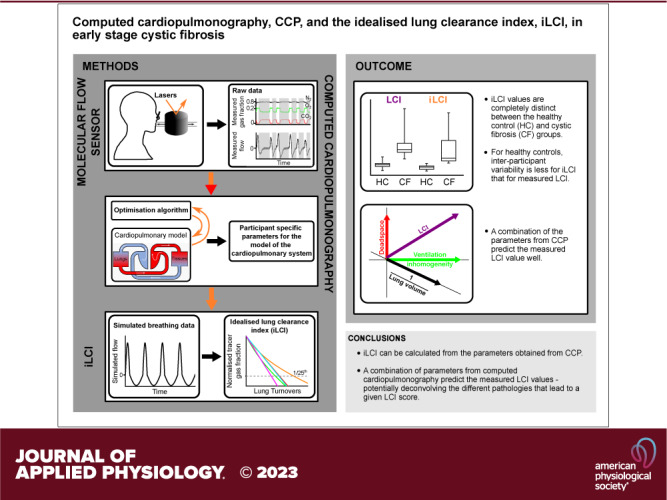

## INTRODUCTION

Cystic fibrosis (CF) is caused by mutations in the gene encoding the cystic fibrosis transmembrane conductance regulator (CFTR) channel. The advent of modulator therapies that both restore the three-dimensional shape of the CFTR channel and potentiate its function has revolutionized the management of many patients with CF (1–3). With the associated improvements in lung function has come an increased need for a more sensitive index of disease than the standard measure of the forced expired volume in one second (FEV_1_). The clinical test that has gained most traction is the multiple-breath washout (MBW) technique (4). This technique has been used to provide primary (5) and secondary (6) outcome measures in clinical trials; a consensus statement for MBW has been developed jointly by the American Thoracic Society and European Respiratory Society (7), and a further working group has examined issues relating specifically to preschool children (8). The main statistic used to summarize the test result is the lung clearance index (LCI_2.5_), which is defined as the number of lung turnovers required to washout either a tracer gas (with air), or N_2_ (with pure O_2_), to 2.5% of the initial starting concentration. Larger values of the LCI_2.5_ are generated by less even ventilation of the lungs, which in CF is generally considered to arise through distal airway narrowing caused by a combination of mucus plugging and inflammation (9).

One of the complications associated with the LCI_2.5_ is that factors other than those that are intrinsic to the lung affect the results obtained. These include, for example, the solubility of the particular tracer gas in the blood and the deadspace of the measurement apparatus (7, 10). A further very important factor is the breathing pattern of the patient, as deeper slower breaths mean that a smaller fraction of each tidal volume is given over to ventilating the deadspace (11, 12). Sometimes this is controlled for by asking the patient to adopt a breathing pattern that targets a particular tidal volume. Often this is larger than a standard tidal volume, which allows additional statistics to be calculated from the slope of the N_2_ plateau during the washout phase. Finally, end-expiratory volume typically varies significantly breath-by-breath in awake, spontaneously breathing patients, and as the single end-expiratory volume immediately preceding the washout (calculated from the amount of N_2_ expired during the washout phase) is used as the divisor for calculating LCI_2.5_, this adds further variation to the test result obtained. To overcome some of these difficulties, a very practical approach is to repeat the test multiple times, and only accept a test result for the LCI_2.5_ when two of the values are within a certain percentage of each other.

The purpose of the present study was to explore the potential of a recently developed technique termed computed cardiopulmonography (CCP) (13) to detect and quantify changes in lung function in early-stage CF. This technique uses a specialist molecular flow sensor (MFS) (14) to obtain highly accurate, highly time-resolved, measurements of the flows of the different respiratory gases at the mouth. These are used to drive a model of the lungs, which includes various aspects of inhomogeneity (15), the blood gas dissociation curves (16), and a model of the circulation and body gas stores (17). The deadspace of the measuring equipment is included within the model, as is the exchange of test gas with the blood. Patient-specific parameters for the lung can be determined by adjusting the model parameters so as to minimize the difference between the output of the model and the data from the patient. CCP has two potential advantages compared with the multi-breath washout technique. First, because the parameters seek to reflect the underlying physiology of the lung, they should not be unduly influenced by test-to-test variations in breathing pattern. Second, because a number of different parameters are simultaneously fit to the data, different parameters of the model have the potential to reflect different aspects of lung disease, for example, bronchiectasis and small airways disease.

As CCP is based on a structural model of the entire cardiopulmonary system, it is possible to simulate a patient’s response to different scenarios. In particular, it is possible to simulate an idealized MBW experiment to calculate an idealized lung clearance index (iLCI_2.5_), where all the extraneous sources of variation have been removed. The apparatus deadspace is set to zero and a hypothetical, completely insoluble, tracer gas is used to simulate the washout. The metabolic rate of the patient is set to a standard value for the patient’s physical characteristics. The breathing frequency is fixed. The tidal volume is then adjusted so as to maintain a standard value for the ideal Pco_2_.

This study provides an initial exploration of CCP in a small group of patients with CF with early-stage lung disease together with a group of healthy control participants. As well as comparing the parameter values between these two groups, the cardiopulmonary model is used to simulate idealized MBW experiments for each participant to generate iLCI_2.5_ values that can be compared with values for LCI_2.5_ that have been measured directly. This provides an opportunity both to explore the influence (or lack thereof) of extrapulmonary variations on the values obtained for the LCI_2.5_ and to gain insight into how different properties of the lung interact to determine the overall value obtained for the LCI_2.5_ within any given individual.

## METHODS

### Participants

Participants were all >18 yr old. They consisted either of healthy controls or of participants with early-stage CF, most of whom had preserved lung function, as defined by an FEV_1_% predicted of >80%.

Recruitment was undertaken at a single specialist clinic. The original intent was to recruit as many adult patients with early-stage CF as possible, along with age- and sex-matched controls, within the time frame available. Unfortunately, the time frame for the experimental study was curtailed by the onset of the COVID-19 pandemic.

Participants visited the lung function testing unit on one occasion. The aim was to undertake a number of N_2_ washouts (at least 2, most commonly 3) using a standard clinical approach for measuring the LCI_2.5_ until two values for LCI_2.5_ had been obtained within 5% of one another. In addition, two N_2_ washouts were undertaken using the MFS to provide data from which values for the iLCI_2.5_ could be calculated.

The study was conducted in accordance with the general principles of the Declaration of Helsinki, with ethical approval obtained from the South Central Oxford A Research Ethics Committee (Reference No. 17/SC/0172). All participants provided written informed consent before undertaking the study.

### Standard LCI_2.5_ Measurements

The MBW was conducted using a free breathing protocol, although the washout phase did not begin until the tidal volumes were established at between 8 and 13 mL/kg ideal body weight. The device used was the Exhalyzer D (eco Medics AG, Switzerland) running version 3.1.6 of the Spiroware software. Subsequent to data collection, an error was recognized arising from cross talk between gas sensors within the Exhalyzer D (18). The MBW results were therefore recalculated using version 3.3.1 of the Spiroware software, which provided a correction for this error. Post hoc quality control was performed in accordance with the European Respiratory Society/American Thoracic Society consensus statement (7) and central over-reading standards (19).

### iLCI_2.5_ Measurements

The process of determining the iLCI_2.5_ fell into three distinct steps detailed below. First, data were collected from a N_2_ washout using the MFS. Second, a set of parameter values were determined for the individual by fitting a model of cardiopulmonary gas exchange to the data from the MFS. Third, the parameters, together with the cardiopulmonary model, were used to simulate an idealized washout under completely standardized conditions using a hypothetical, completely insoluble, gas. The iLCI_2.5_ was calculated from this idealized washout. The overall process of obtaining highly accurate measurements of gas flow, and then using the data to estimate parameter values in a computational model of the lungs and body gas stores, we term computed cardiopulmonography (CCP).

#### Molecular flow sensor.

The MFS is a bespoke instrument designed to provide highly accurate, highly time-resolved, measurement of the flows of the individual gas species that are involved in breathing. The accuracy of these flows is such that it makes it possible to use them to drive computational models of the lung over many minutes without significant integration error (integration error would cause the model lung to inflate or deflate to abnormal volumes).

The MFS has been described in detail elsewhere (14). In brief, it uses laser absorption spectroscopy to determine the CO_2_, O_2_, and water vapor contents within the main respired gas stream every 10 ms. The pressure and temperature of the respired gas are also recorded, and from these and the measured gas contents, the N_2_ content (to include Ar) can be calculated. Flow is measured by the pressure drop across two stainless steel meshes, one either side of the gas analysis plane. The in-line measurement of the gas contents together with temperature and pressure, enable both the density and viscosity of the gas to be calculated every 10 ms. Knowledge of these physical characteristics of the flow makes possible the accurate translation of the pressure drop into flow.

#### Cardiopulmonary model and parameter estimation process.

**Figure 1.**
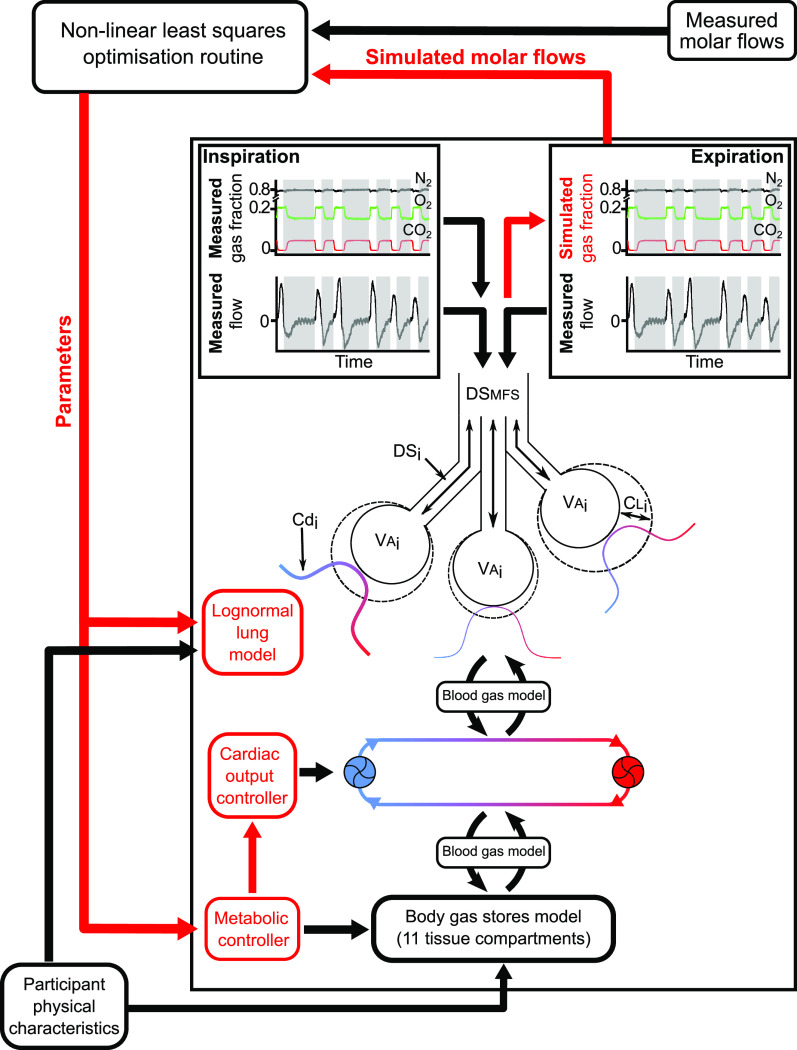
Schematic illustrating model and parameter estimation technique. There are three submodels, the first is a model for the blood gas dissociation curves, the second is a model of the circulation and body gas stores, and the third is a model of the lung that includes inhomogeneity (three of the total 125 compartments are illustrated). The size of the body gas stores and some of the initial parameter predictions are determined by the participant’s physical characteristics. The model is driven through metabolic consumption of O_2_ and production of CO_2_. This, along with body size, determines cardiac output. The respiratory flows recorded by the molecular flow sensor drive the ventilation of the lungs, and the inspiratory composition of the gas is set to match that recorded with the molecular flow sensor. Simulated expiratory compositions are then calculated during the execution of the model. These give rise to simulated molar flows for each gas species during expiration and can be compared with those measured by the molecular flow sensor. A nonlinear optimization routine then adjusts the parameters between successive runs of the model so as to minimize the error between the simulated and experimentally recorded molar flows for the different gases during expiration. Cd_i_, vascular conductance for the i^th^ lung unit; CL_i_, compliance for the i^th^ lung unit; DS_i_ deadspace for the i^th^ lung unit; DSMFS apparatus deadspace for the molecular flow sensor; VA_i_, alveolar volume for the i^th^ lung unit.

The cardiopulmonary model used in this study is a combination of three published models. The first is a physicochemical model of blood that effectively provides dissociation curves for CO_2_ and O_2_ that include the Bohr and Haldane effects (16). The second model is a multicompartment model of the lung designed to model inhomogeneity within the lung (15). The third is a model of the circulatory and body gas stores that accounts for the recirculation of blood back to the lungs and therefore for the loading and unloading of the body gas stores (17). How these three models have been combined for the purposes of parameter estimation from data gathered by the MFS has been described in more detail in a recent study of lung physiology in patients who had recovered from COVID-19 (13), and is illustrated in Fig. 1.

In brief, the governing equations of the model are built on the principle of mass balance, and the model is driven through the consumption of O_2_ and production of CO_2_ in the tissue compartments. The cardiac output in the model is determined principally by metabolism, and this delivers the venous blood to the lungs. The lung model consists of 125 compartments and the distribution of blood flow and ventilation to these compartments is determined by a bivariate log-normal distribution for standardized vascular resistance and compliance across the lung (the absolute values for vascular resistance and lung compliance are not necessary for distributing the blood flow and ventilation correctly). The ventilation of the lungs during both inspiration and expiration is generated to match exactly the total respiratory flow as recorded by the MFS. The inspired gas composition is also provided to the model lung exactly as measured by the MFS, and this is sufficient to allow the model to calculate the gas fractions throughout expiration. The parameter estimation process works by adjusting the parameters of the model so that these calculated expired gas fractions match as closely as possible to those recorded by the MFS.

Prior to any run of the model, the 125-compartment model lung has to be built in accordance with the parameters specifying the end-expiratory alveolar volume, deadspace volume, the standard deviations associated with the standardized lung compliance and vascular resistance, and a standard deviation for the standardized deadspace. After this, the size of the gas stores throughout the model requires initialization. This is achieved for the specified metabolic rate by use of a standardized breathing pattern where the magnitude of the tidal volume is adjusted until the ideal Pco_2_ for the lung matches the parameter value for this at the start of simulation.

In general, parameters can either be measured directly from the patient, or be taken from the literature, or be fitted to the individual, and the choices made can vary depending on the purposes of the study and the input stimulus employed. For most studies, including this one, patient-specific values for height, weight, age, and sex are used so that the sizes of the body gas stores are calculated correctly, and so that body size can be taken into account when setting the initial guesses for parameters such as alveolar volume at functional residual capacity. For other parameters, such as the concentration of hemoglobin and various ions in blood, values have been taken from the literature. In this study, where the input stimulus is the N_2_ washout, we estimated a total of eight parameters. These are listed in Table 1. Five of the parameters relate specifically to the lung and include the standard deviations for the natural logarithm of the standardized lung compliance (σlnCl) and the standard deviation for standardized deadspace (σVD). In this study, the standard deviation for the natural logarithm of the standardized vascular resistance (σlnCd) was assumed to be an additional 0.3 units greater than the patient’s value for σlnCl. The other three parameters specify the metabolism through the rate of oxygen consumption and the respiratory quotient, and the initial loading of the body gas stores with CO_2_ through the starting value for the ideal Pco_2_ of the lung.

**Table 1.** Parameters estimated within the fitting process

DS, L (BTPS)	Deadspace (DS) volume at functional residual capacity (FRC).
Cvd	Fractional expansion of deadspace relative to fractional expansion of alveolar space.
σVd	Standard deviation for the standardized deadspace.
Va, L (BTPS)	Alveolar volume at FRC (FRC = DS + VA).
σlnCl	Standard deviation for the natural logarithm of the standardised lung compliance.
V̇o_2_, L (STPD)/min	Oxygen consumption.
R	Respiratory quotient.
Pi_CO_2__, kPa	Ideal Pco_2_ at initialization of model.

The model was predominantly coded in MATLAB (R2022a, MathWorks Inc, Natick, MA) but with two small, numerically intensive, routines coded in C++. The model and parameter estimation process were run on the ARCUS high-performance computer system at the University of Oxford on a single multicore (≥ 16 core) processor. The fitting process generally took <1 h to complete. For each dataset, the fitting process was run four times from different randomized starting positions to check that each fit converged on the same minimum. Occasionally, this was not the case, and then the number of fits could be increased until several had converged on a common lowest minimum.

#### Calculation of the idealized lung clearance index.

The iLCI_2.5_ is a simulated value for the LCI_2.5_ calculated from the cardiopulmonary model under conditions when all extrapulmonary influences on the LCI_2.5_ have been standardized. The parameters relating to the lung in the cardiopulmonary model were those estimated for the individual by CCP.

The parameters replaced with standard values were as follows: a standard value for the individual’s oxygen consumption (V̇o_2_), and from this the cardiac output (Q̇), was determined from the individual’s height, mass, sex, and age following the method employed in the model of the circulatory and body gas stores (17). A respiratory quotient (R) of 0.8 was assumed to calculate the individual’s carbon dioxide production (V̇co_2_). A standard value of 5.3 kPa was chosen for the ideal Pco_2_. Finally, a respiratory rate (RR) was assumed, the choice of which is explored in the results. Once the RR rate has been chosen, the associated tidal volume that generates the ideal Pco_2_ can be found by an iterative process (in general, specifying any two of the ideal Pco_2_, RR, and tidal volume will determine the third).

With five exceptions, the initialization and running of the cardiopulmonary model followed the same procedure as that used for the parameter estimation process. The five exceptions were *1*) the apparatus deadspace was removed so that it had no influence on the iLCI_2.5_; *2*) the initial inspired gas was air with 1% of the N_2_ replaced by the hypothetical, completely insoluble, tracer gas; *3*) the constant, fixed ventilatory pattern used during the initialization was used throughout the simulation (during model fitting, the participant’s recorded respiratory flow was used to drive the model); *4*) the washout phase of the simulation was initiated by removing the hypothetical tracer gas from the air and replacing it with N_2_; and *5*) the composition of the pulmonary arterial blood was held constant (no recirculation).

The synthetic respiratory flow pattern consisted of two half sine waves, one for inspiration and the other for expiration. The duration of inspiration relative to expiration was set in the ratio 1:3. The volume [standard temperature and pressure, dry (STPD)] of inspiration slightly exceeded the volume of expiration, based on the difference between V̇o_2_ and V̇co_2_, so as to maintain a constant end-expiratory volume (at functional residual capacity) throughout the simulation.

To calculate the iLCI_2.5_, after the onset of the washout phase, the end-tidal values for the tracer gas were interpolated to identify the time at which the concentration has fallen to 2.5% of its starting value. This time is then converted into the number of lung volume turnovers (the LCI_2.5_) by dividing the total volume of gas expired during the washout phase up to this time by the FRC of the lung.

### Statistical Comparisons

For a comparison of means between control participants and those with CF, an unpaired Student’s *t* test was used where the variances were not significantly different, and Welch’s *t* test was used in cases where the variances were significantly different. Variance ratios were compared using an *F* ratio test. Multiple linear regression was used to explore the significance of potential predictors of the LCI_2.5_. Statistical significance was assumed at *P* < 0.05.

## RESULTS

### Participants

A total of 25 participants were recruited, 14 were healthy controls (HC, numbered 1–14) and 11 were patients with CF (numbered 15–25), most of whom had a preserved lung function defined as an FEV_1_ > 80% predicted. Table 2 lists their physical characteristics.

**Table 2.** Participant characteristics

		Participants with Cystic Fibrosis			
	Healthy Control Participants	Young	Old	Total	Overall
Number of participants	14	7	4	11	25
Female (%)	7 (50)	6 (86)	0 (0)	6 (55)	13 (52)
Age, yr	21.9 ± 1.3	22.4 ± 2.8	42 ± 8.3	29.5 ± 11.1	25.3 ± 8.2
Age range, yr	21–26	19–28	35–54	19–54	19–54
Height, m	1.71 ± 0.10	1.63 ± 0.10	1.72 ± 0.05	1.66 ± 0.09	1.69 ± 0.10
Weight, kg	67.9 ± 13.7	64.2 ± 13.9	70.6 ± 7.6	66.5 ± 12.0	67.3 ± 12.6
BMI, kg/m^2^	23.2 ± 4.0	24.1 ± 4.2	24.0 ± 22.1	24.0 ± 3.4	23.6 ± 3.3
Respiratory rate, min^−1^	14.3 ± 4.1	15.1 ± 5.2	14.6 ± 2.7	15.0 ± 4.3	14.6 ± 4.1
Tidal volume, mL/kg (ideal body weight)	8.3 ± 1.7	8.3 ± 1.2	10.1 ± 0.7	8.9 ± 1.4	8.5 ± 1.5

Healthy control and combined cystic fibrosis groups differed significantly for age (*P* = 0.047, Mann–Whitney test). All other variables were not significantly different between the two groups (Student *t* test). Means ± SD. BMI, body mass index.

The early termination of the experimental phase of this study due to the onset of the COVID-19 pandemic resulted in less good age matching between the HC and CF groups than was originally intended. Seven participants of the CF group were young and well matched with the HC group, all of whom were also young. However, four participants of the CF group were older, and for these, there was a lack of age-matched controls. Table 2 therefore illustrates the physical characteristics for these two groups of participants with CF separately as well as for the combined CF group. Where this may be of interest in later figures, a distinction is also drawn between the two groups of patients with CF.

All participants completed at least one successful measurement with the MFS. For three participants (1 HC and 2 CF), there were no corresponding LCI_2.5_ data because they did not pass quality control (7, 19) and/or there was insufficient time to gain two satisfactory washouts.

### Estimation of Model Parameters by Computed Cardiopulmonography

**Figure 2.**
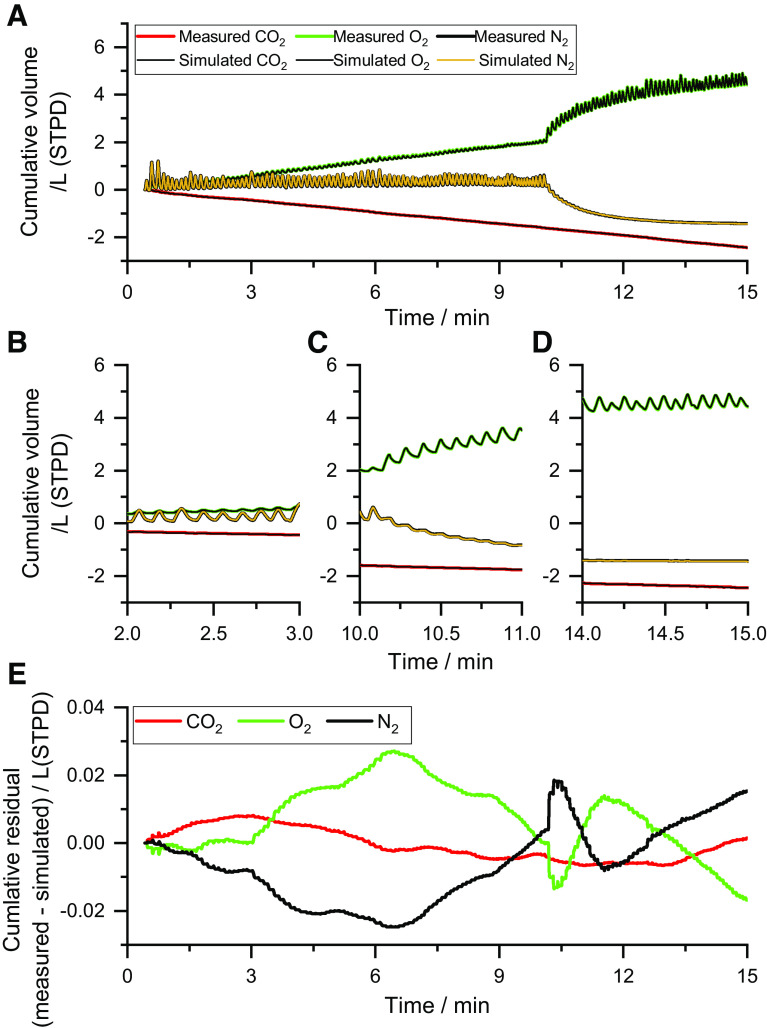
Example of the fit of the model to the data. *A*: measured and model-simulated cumulative tidal flows for the different gas species at the mouth. The switch from breathing air to breathing pure O_2_ occurs at ∼11 min. The model responses are essentially overlaid on the data. *B–D*: 1-min periods on an expanded scale from the record in *A*, illustrating the results during the air-breathing phase, and early and late in the N_2_ washout phase. *E*: cumulative residuals (measured minus simulated) illustrating the residual error in the model fit for the gas exchange data. Data are from participant number 17, who was a 22-yr-old female with cystic fibrosis. The sum of squared errors for the fit was 0.078 (L/s)^2^. STPD, standard temperature and pressure, dry.

Figure 2 illustrates an experimental record obtained with the MFS together with the best-fit tidal flows for the different gas species. Points to note are the overall trends for CO_2_ and O_2_ associated with metabolism, and the absence of such a trend for N_2_ during the air-breathing phase. Following the switch to breathing 100% O_2_ at ∼10 min, there was a reduction in the tidal flow of N_2_ associated with its washout from the lung together with a corresponding increase in the tidal flow of O_2_ as it washed into the lung. The model fits to these flows were extremely good, and the deviations are poorly discernible in Fig. 1*A* due to data point overlap. Figure 1, *B–D*, illustrates 1-min sections of the record on an expanded time scale. Finally, Fig. 1*E* illustrates the deviations between model and data as a plot of the cumulative residuals against time. For this participant, the sum of squared residuals was 0.078 (L/s)^2^, which compares with overall averages of 0.235 ± 0.178 (L/s)^2^ (means ± SD) for the HC group and 0.224 ± 0.103 (L/s)^2^ for the CF group.

**Figure 3.**
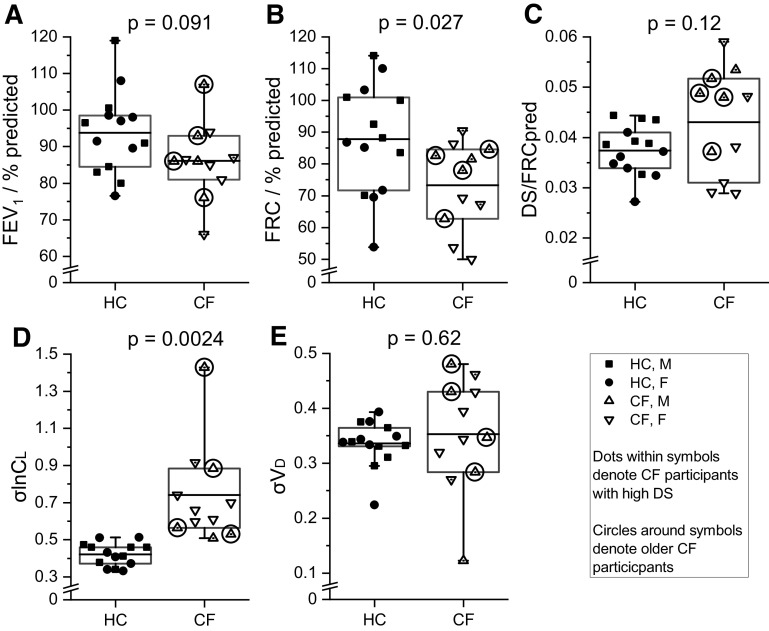
Comparison for selected parameters between the healthy control (HC) and cystic fibrosis patient (CF) groups. *A*: plot of the measured forced expiratory volume in one second (FEV_1_) % predicted for the HC (*n* = 14) and CF (*n* = 9) groups; *n* represents number of participants. *B–E*: plots showing parameters determined by computed cardiopulmonography for the HC (*n* = 14) and CF (*n* = 11) groups. Functional residual capacity (FRC) has been plotted as a percentage of the predicted value. Data are shown for each individual, as well as boxes illustrating the interquartile range, together with a horizontal line indicating the mean value. The whiskers illustrate the spread of data outside of the interquartile range and extend to the lowest or highest data point that is within three times the interquartile range of the lower or upper border of the box. Data from the older (>30 yr) participants with CF are indicated by circles surrounding the symbols. Data from participants with CF with enlarged deadspace (*C*) are indicated by the use of a dot within the symbols. The statistical significance of differences between the groups was determined using a Welch *t* test. DS, end-inspiratory deadspace; CF, cystic fibrosis; F, female; FRCpred, predicted FRC; σlnCl, standard deviation for the natural logarithm of the standardized lung compliance; HC, healthy control; M, male; σVD, standard deviation for the standardized deadspace.

Figure 3*A* illustrates the values for FEV_1_% predicted, which were not significantly different between the HC and CF groups. The remainder of Fig. 3 compares the CCP model parameter values obtained for HC and participants with CF. In general, these parameter values are the mean values from the two repeats of the CCP protocol in each participant and their within-participant coefficients of variation were: FRC (% predicted), 5.7%; end-inspiratory deadspace (DS) relative to predicted FRC, 6.7%; σlnCl, 6.9% and σVD, 7.1%. FRC is shown in Fig. 3*B* as % predicted to control for physical characteristics, and DS is shown in Fig. 3*C* as a fraction of predicted FRC for the same reason. The remaining two panels illustrate data for the inhomogeneities σlnCl and σVD across the lung. The CF group had values for FRC % predicted that were significantly smaller than for the HC group (Fig. 3*B*). For all the other remaining variables (DS/FRCpred, σlnCl, and σVD), the spread of values for the participants with CF was greater than for the HC participants (*F* = 5.9, *P* < 0.002; *F* = 23.9, *P* < 10^−6^; *F* = 8.2, *P* < 0.001; for DS/FRCpred, σlnCl, and σVD, respectively).

### Estimation of the iLCI_2.5_ and Repeatability of Measurements

**Figure 4.**
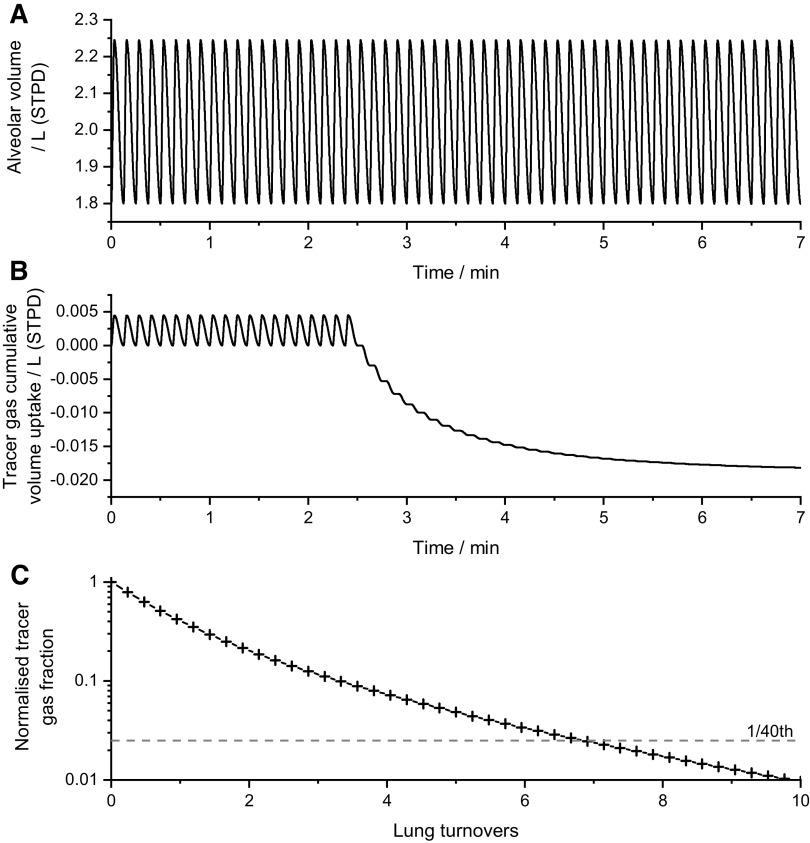
Calculation of the idealized lung clearance index from the simulated tracer gas washout protocol. *A*: alveolar volume against time, throughout the simulated protocol, showing the regular breathing pattern. *B*: cumulative volume uptake of the theoretical tracer gas against time. This oscillates to and from zero for the first 2.5 min as it is breathed in and out, after which it is progressively washed out by air (which contains no tracer gas). *C*: plot during the washout phase of the tracer gas fraction at the mouth (normalized relative to the starting fraction) against the cumulative expired volume.

Figure 4 illustrates the estimation of the iLCI_2.5_ for the same participant whose experimental record is shown in Fig. 1. This simulation was performed with a respiratory rate of 8 breaths per min. Note the complete regularity of the lung volume record and also the complete stability of end-expiratory volume. The end-tidal record for the tracer gas shows a smooth monotonic decline during the washout and it is easy to identify when the tracer gas has declined to 2.5% of its original value.

**Figure 5.**
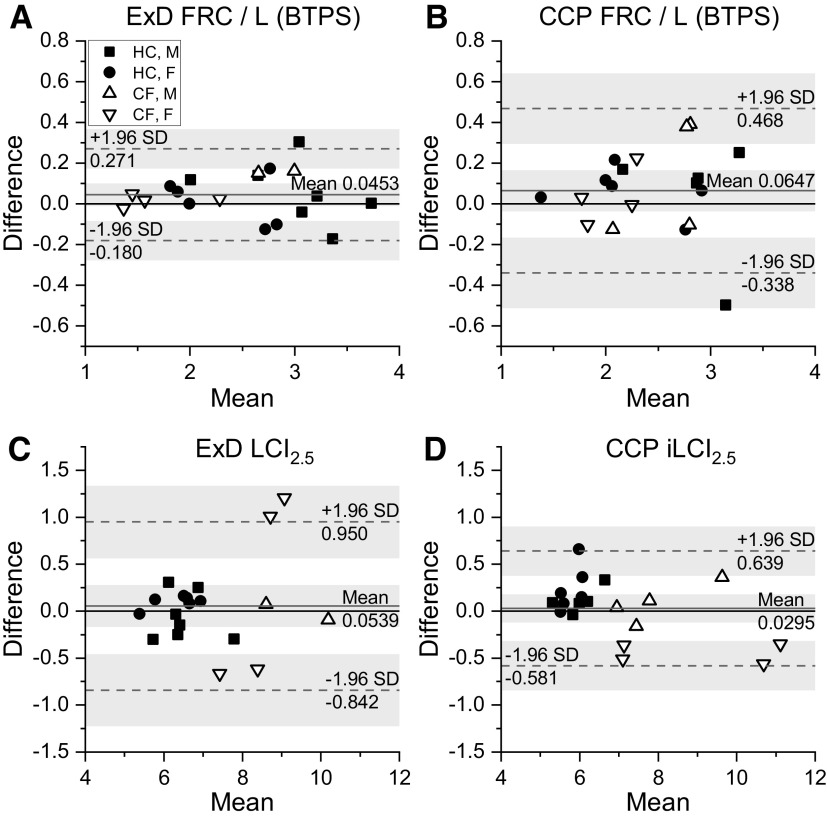
Repeatability of measurements made with the Ecomedics Exhalyzer D (ExD) device and with computed cardiopulmonography (CCP). *A* and *B*: repeatability of FRC measurement made with the ExD (HC, *n* = 13; CF, *n* = 6) and with CCP (HC, *n* = 11; CF, *n* = 8), respectively; *n* represents number of participants. *C* and *D*: repeatability of the lung clearance index (LCI_2.5_) made with the ExD and the idealized-LCI_2.5_ (iLCI_2.5_) made using CCP, respectively. Patient numbers and statistical comparisons, as for *A* and *B*. Shaded areas indicate the 95% confidence intervals for the mean and for the values for ±1.96 SD. CCP, computed cardiopulmonography; CF, cystic fibrosis; FRC, functional residual capacity; HC, healthy control.

Figure 5 contains data from only those participants for whom there were two successful washouts within a given technique that could be compared. The figure illustrates the repeatability of the FRC and LCI_2.5_ measurements for both the commercial Exhalyzer D system (Fig. 5, *A* and *C*) and the CCP iLCI_2.5_ approach (Fig. 5, *B* and *D*). The FRC measurements for the Exhalyzer D system were more repeatable than those obtained using CCP (*F* ratio = 3.2, *P* < 0.01). In contrast, the iLCI_2.5_ measurements from CCP appeared more repeatable than the LCI_2.5_ measurements from the commercial Exhalyzer D system, although this did not reach statistical significance (*F* ratio = 2.2, *P* = 0.056). This last comparison needs some qualification because, as part of the protocol, pairs of values for the standard LCI_2.5_ were only accepted if they were within 5% of one another, whereas this step was not undertaken for the CCP iLCI_2.5_. Indeed, a number of the participants with CF with the highest values for the LCI_2.5_ could not be included in the calculation of the SD for the standard LCI_2.5_ as there were no two measurements within 5% of one another.

### Effect of Respiratory Rate

**Figure 6.**
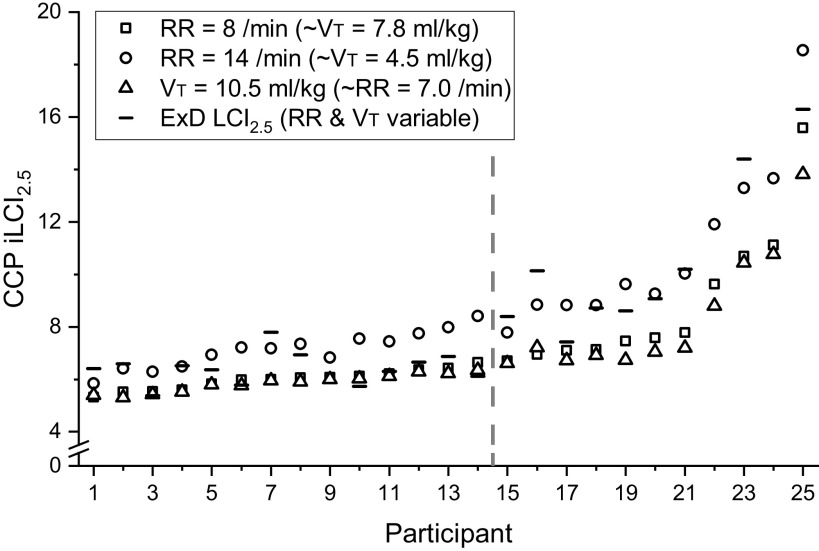
Effect of varying the simulated breathing pattern on the values calculated for the idealized lung clearance index, iLCI_2.5_. Participant numbering is ordered by value of iLCI_2.5_ at a respiratory rate of 8 breaths per minute. Vertical broken line separates the HC participants to the left, and participants with CF to the right. The tidal volume of 10.5 mL/kg of ideal body weight is the midpoint of the range (8–13 mL/kg) considered acceptable for the standard clinical measurement of LCI_2.5_. Also shown are the experimentally determined values for LCI_2.5_ from the standard washout procedure where the breathing frequency differed between participants. There was a positive correlation (*r* = 0.46, *P* = 0.031) between the value for the LCI_2.5_ relative to the iLCI_2.5_ (at 8 breaths/min) and the participant’s breathing frequency during the LCI_2.5_ measurement. CF, cystic fibrosis; HC, healthy control; RR, respiratory rate; VT, tidal volume.

Figure 6 compares the iLCI_2.5_ values at a respiratory rate of 8 breaths per minute with those obtained at a respiratory rate of 14 breaths per minute. For all participants, the more rapid shallow breathing increased the fraction of the ventilation that is deadspace and therefore increased iLCI_2.5_. Also shown are the iLCI_2.5_ values when, in place of the set breathing frequency, the tidal volume was set to 10.5 mL/kg, which is in the middle of the range targeted by the lung function testing laboratory at the Brompton. These values were similar, though perhaps a little lower at higher values for iLCI_2.5_, to those obtained with the breathing frequency set to 8 breaths per min and this informed our choice of breathing frequency for calculating the iLCI_2.5_. Note that the numbering of the participants in this study has been ordered by their iLCI_2.5_ at 8 breaths per min and also that HC participants all had iLCI_2.5_ values below those for all the participants with CF. Finally, Fig. 6 also includes the measured values for LCI_2.5_, which vary in position relative to those for the iLCI_2.5_. These values, relative to the iLCI_2.5_ at 8 breaths per min, correlate positively (r = 0.46, *P* = 0.031) with participant’s breathing frequency during the LCI_2.5_ measurement.

### Comparison of LCI_2.5_ and iLCI_2.5_

**Figure 7.**
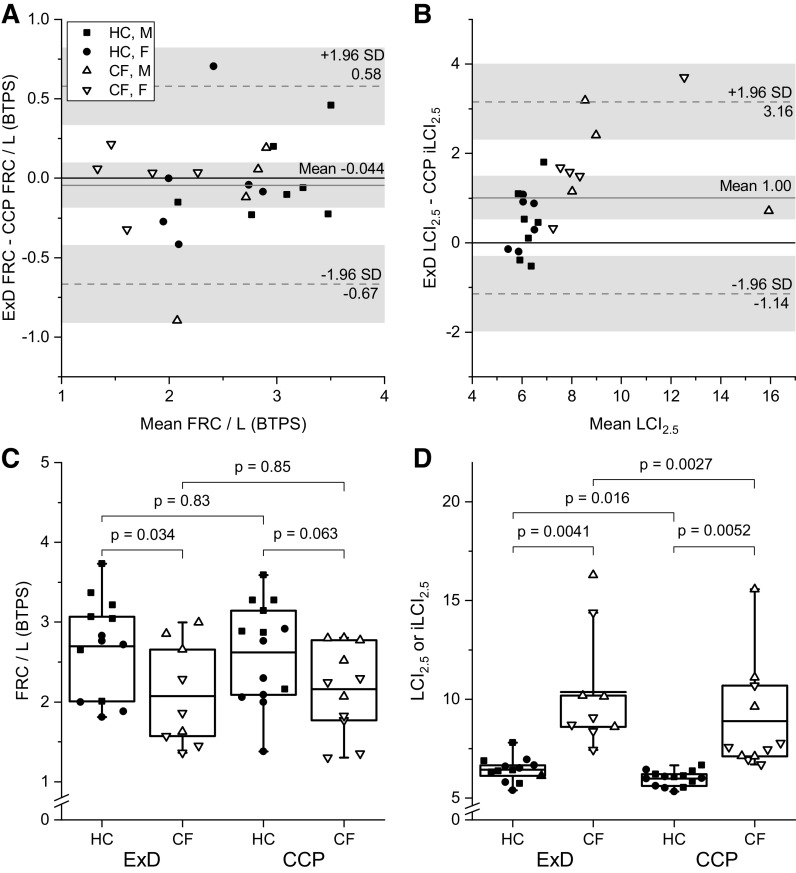
Comparison of FRC and LCI_2.5_ measured using ExD with FRC and iLCI_2.5_ determined using CCP. *A*: comparison of FRC values between the two techniques (HC, *n* = 13; CF, *n* = 9). *n* represents number of participants. *B*: comparison of iLCI_2.5_ with LCI_2.5_ (HC, *n* = 13; CF, *n* = 9). *C*: box and whisker plots as for Fig. 2 comparing FRC values for HC and CF groups from ExD (HC, *n* = 13; CF, *n* = 9) and from CCP (HC, *n* = 14; CF, *n* = 11). *D:* box and whisker plots comparing values for ExD LCI_2.5_ with those for CCP iLCI_2.5_ for HC and CF groups. Participant numbers as for *C*. CCP, computed cardiopulmonography; CF, cystic fibrosis; ExD, Ecomedics Exhalyzer D; FRC, functional residual capacity; iLCI_2.5_, idealized lung clearance index; HC, healthy control; LCI_2.5_, lung clearance index.

Figure 7 provides a comparison between the FRC and LCI_2.5_ values obtained with the Exhalyzer D and those obtained using the CCP iLCI_2.5_ approach. Figure 7, *A* and *C*, illustrates that the two techniques give very similar results for FRC. In contrast to this, values for the iLCI_2.5_ were generally lower than for the LCI_2.5_ (Fig. 7, *B* and *D*), and this difference became more pronounced at higher values for LCI_2.5_. This was most likely caused by the relatively low respiratory rate chosen for the iLCI_2.5_ calculation. Both techniques discriminated well between the two groups of participants. For the HC participants, the SD for the LCI_2.5_ measurements was 0.61, and for the iLCI_2.5_ measurements, it was 0.38 (F-ratio = 2.57, *P* < 0.05).

### Components Contributing to the Overall iLCI_2.5_ Value

**Figure 8.**
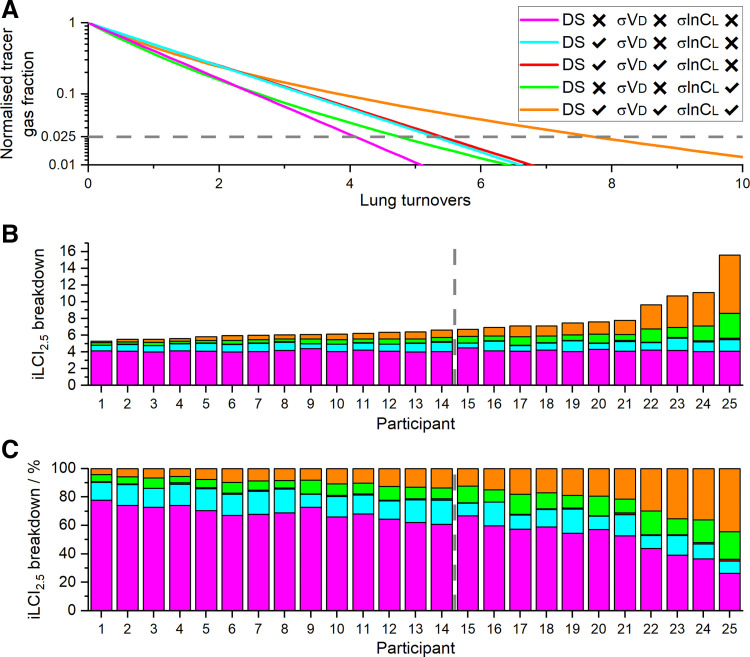
Breakdown of the contributions of different aspects of lung inhomogeneity to overall values for iLCI_2.5_. *A*: example of the simulated washout profiles, showing normalized tracer gas fraction against the expired volume measured in lung turnovers. The different records are for *1*) a homogenous lung with no deadspace (DS); *2*) a lung with DS but without either deadspace inhomogeneity (σVD) or ventilation inhomogeneity (σlnCL); *3*) a lung with DS and σVD, but without σlnCL; *4*) a lung with σlnCL but with no DS; and *5*) a lung with all inhomogeneities present. Horizontal broken line indicates 1/40^th^ of starting fraction for tracer gas. *B* and *C*: stacked bar charts illustrating the contribution of each aspect of lung inhomogeneity to the total iLCI_2.5_ for each participant in absolute terms in *B*, and in percentage terms in *C*. Participants have been numbered in ascending order of iLCI_2.5_. The older participants with CF are numbers 19, 21, 22, and 25. The vertical line indicates the division between the HC group (*participants 1–14*) to the left, and the CF group (*participants 15–25*) to the right. CF, cystic fibrosis; HC, healthy control.

Figure 8 explores the components contributing to the overall value for the iLCI_2.5_. Figure 8*A* illustrates the methodology. Figure 8, *B* and *C*, provides stacked bar charts for each participant illustrating the contributions of different factors to their overall iLCI_2.5_ value. There are two particular points of note. First, the sum of the individual contributions of parameters to the iLCI_2.5_ fell well short of the effect when all the parameters were included in the model together. This interactive effect became an increasingly large fraction of the total iLCI_2.5_ as the absolute value of the iLCI_2.5_ increased. Second, some participants with similar values for the iLCI_2.5_ had very different underlying physiology. For example, for *participants 16, 17*, and *18*, all had a similar iLCI_2.5_, *participant 17* had less deadspace but more ventilation inhomogeneity than either *16* or *18*. The same can be observed for *participant 20* in relation to *participants 19* and *21*.

**Figure 9.**
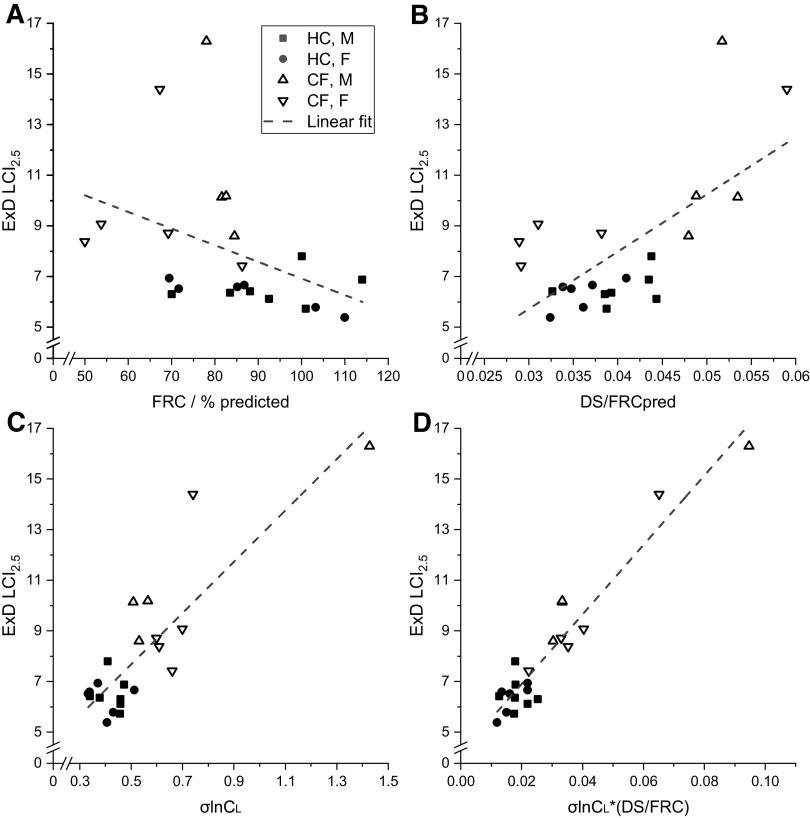
Relationship between ExD LCI_2.5_ and selected model variables from CCP. *A*: LCI_2.5_ vs. FRC % predicted. *B*: LCI_2.5_ vs. DS/FRCpred. *C*: LCI_2.5_ vs. σlnCL. *D*: LCI_2.5_ vs. σlnCL*(DS/FRC). Participants: HC, *n* = 13; CF, *n* = 9. Broken lines illustrate simple linear regressions, significant for *B–D*, but not *A*. Multiple linear regression was significant for σlnCL*(DS/FRC) (*F* = 147, *P* < 0.001) and DS/FRCpred (*F* = 7.5, *P* < 0.02), indicating interaction between different aspects of lung function is the most important overall determinant of LCI_2.5_. CCP, computed cardiopulmonography; CF, cystic fibrosis; DS, deadspace; ExD, Ecomedics Exhalyzer D; FRC, functional residual capacity; HC, healthy control; LCI_2.5_, lung clearance index.

Figure 9 explores the degree to which the CCP parameters could be used to predict the standard LCI_2.5_ measurement obtained using the Exhalyzer-D. Individually, DS/FRCpred and σlnCl, but not FRC % predicted, were significantly correlated with LCI_2.5_ (although the relationships may differ between the HC and CF groups). Following this, a multiple linear regression was conducted that included each of these terms plus an interactive term of the form σlnCl*(DS/FRC). Nonsignificant covariates were then removed sequentially, least significant first, until only the significant covariates remained. At the end of this process, the only significant covariates were the interactive term [σlnCl*(DS/FRC)] and DS/FRCpred, and of these, the interactive term was by far the most important predictor [F-ratio = 147, *P* < 0.001, for σlnCl*(DS/FRC) vs. F-ratio = 7.5, *P* < 0.02, for DS/FRCpred]. The relationship for LCI_2.5_ versus σlnCl*(DS/FRC) is shown in Fig. 9*D*.

## DISCUSSION

This study explored the use of CCP to assess lung function in adults with early-stage (preserved FEV_1_) CF. The main findings were *1*) that their lungs expanded and contracted less evenly than the control participants, as evidenced by an increase in σlnCl, and *2*) that the parameters relating to deadspace (DS/FRCpred and σVD) were more variable, with some participants with CF having noticeably higher values than control participants. iLCI_2.5_ values that were simulated under standardized conditions from the CCP parameters distinguished clearly between participants with CF and HC participants, as did experimentally measured values for LCI_2.5_ using a standard clinical technique. LCI_2.5_ values were generally larger than iLCI_2.5_ values, and this effect was greater in those participants who breathed at higher respiratory frequencies during the clinical measurement. A sensitivity analysis revealed a significant interactive effect between the different CCP parameters in determining the iLCI_2.5_ value, and this effect increased with increasing values of iLCI_2.5_. Finally, it was found that an interaction term between the parameters of the form σlnCl*(DS/FRC) was a good linear predictor of the LCI_2.5_. These latter findings raise the possibility that CCP could potentially be used to “untangle” different aspects of lung disease in CF that are currently all consolidated within a single LCI_2.5_ value.

In patients with chronic obstructive pulmonary disease (COPD), Verbanck et al. (20) compared directly measured MBW curves with those simulated from ventilation inhomogeneity measured by computed tomography. They found that the ventilation inhomogeneity assessed radiologically contributed a little less than half of the total increase in LCI_2.5_ with COPD. They attributed the other half of the increase in LCI_2.5_ to a reduction in alveolar mixing efficiency. This reduction in alveolar mixing efficiency may well correspond to the increase in anatomical deadspace in COPD that was observed by Mountain et al. (15) when fitting the log-normal lung model to patients. If so, then the findings of Verbanck et al. would seem very consistent with the current results, namely that deadspace is an important interactor with ventilation inhomogeneity in determining the LCI_2.5_. Indeed, this is further supported by a study demonstrating that LCI_2.5_ values increased with the number of bronchial segments affected by bronchiectasis (21). The authors noted that the clinical implication of their study was that the extent of bronchiectasis increased the lowest possible LCI_2.5_ value to which an individual patient could be reversed after treatment. This is a problem that most likely could be avoided if the CCP parameters were used in place of the LCI_2.5_.

The cardiopulmonary model in the present study was based around the properties of the healthy lung. As such, there is a significant possibility that it will become a less good model as a patient’s lungs become progressively more diseased. One of the model’s assumptions is that flow can be apportioned in a constant manner between the different alveolar units throughout inspiration and expiration. The lung units thus differ in terms of their static compliance, and this assumption has a significant computational advantage in that the lung units can be treated as independent during breathing. An alternative approach is to model the airway in terms of resistances, and if these are sufficiently great and the associated time constants for emptying sufficiently long, then this can instead result in asynchronous emptying of the lung units (22). In asthma, such asynchronous emptying has been suggested as a potential mechanism to explain the increase in the phase III slope for N_2_ during a MBW (22). At present, it is hard to tell how well the current model will perform in patients with more severely diseased lungs. However, our overall objective has always been to focus on improving measurement technologies for early or mild disease where the assessment of lung function with traditional techniques such as FEV_1_ has proved least satisfactory.

In summary, this study has demonstrated that it may be possible to extract substantially more information from a MBW measurement if a very accurate measurement technology is coupled with a modeling approach to estimate patient-specific parameters for a mechanistic model of lung function. This approach has provided some insight into the factors underlying the standard clinical measure of the LCI_2.5_, and it may also hold promise for a more multidimensional assessment of lung function in patients with CF.

## DATA AVAILABILITY

Data will be made available upon reasonable request.

## GRANTS

This work was supported by grants from the Engineering and Physical Sciences Research Council (EP/T001186/1), the Cystic Fibrosis Trust (Venture and Innovation Award 068), and the National Institute for Health Research (NIHR) Oxford Biomedical Research Centre (BRC). D.S. was supported by a Clarendon Scholarship from the University of Oxford. J.L.R. was supported by an Engineering and Physical Sciences Research Council studentship. J.C.D, C.J.S., and C.S. were supported by the NIHR through the Imperial Biomedical Research Centre, the Brompton Clinical Research Facility, and a Senior Investigator Award (to J.C.D.).

## DISCLAIMERS

The views expressed are those of the authors and not necessarily those of the NHS, the NIHR, or the Department of Health.

## DISCLOSURES

Oxford University Innovation, a wholly owned subsidiary of the University of Oxford, holds/has filed patents relating to the background IP for the technology. J.H.C., G.A.D.R., and P.A.R. have an interest in one or more patents. The European Cystic Fibrosis Society’s (ECFS) LCI Core Facility received start-up funding from the ECFS and has supported clinical trials in CF sponsored by a number of commercial agencies. C.S., C.J.S., and J.C.D. are involved with this facility. None of the other authors has any conflicts of interest, financial or otherwise, to disclose.

## AUTHOR CONTRIBUTIONS

J.C.D., G.A.D.R., and P.A.R. conceived and designed research; J.L.R., N.M.J.S., C.S., C.J.S., J.H.C., and G.R. performed experiments; D.S., J.L.R., C.S., C.J.S., C.J.F., and G.R. analyzed data; D.S. and P.A.R. interpreted results of experiments; D.S. and J.L.R. prepared figures; P.A.R. drafted manuscript; D.S., J.L.R., C.S., J.C.D., G.A.D.R., P.A.R., and N.P.T. edited and revised manuscript; P.A.R. approved final version of manuscript.
